# Path Planning Method for UUV Homing and Docking in Movement Disorders Environment

**DOI:** 10.1155/2014/246469

**Published:** 2014-06-22

**Authors:** Zheping Yan, Chao Deng, Dongnan Chi, Tao Chen, Shuping Hou

**Affiliations:** ^1^College of Automation, Harbin Engineering University, Harbin 150001, China; ^2^Beijing Institute of Space Mechanics & Electricity, Beijing 100094, China; ^3^College of Mechanical and Electrical Engineering, Harbin Engineering University, Harbin 150001, China

## Abstract

Path planning method for unmanned underwater vehicles (UUV) homing and docking in movement disorders environment is proposed in this paper. Firstly, cost function is proposed for path planning. Then, a novel particle swarm optimization (NPSO) is proposed and applied to find the waypoint with minimum value of cost function. Then, a strategy for UUV enters into the mother vessel with a fixed angle being proposed. Finally, the test function is introduced to analyze the performance of NPSO and compare with basic particle swarm optimization (BPSO), inertia weight particle swarm optimization (LWPSO, EPSO), and time-varying acceleration coefficient (TVAC). It has turned out that, for unimodal functions, NPSO performed better searching accuracy and stability than other algorithms, and, for multimodal functions, the performance of NPSO is similar to TVAC. Then, the simulation of UUV path planning is presented, and it showed that, with the strategy proposed in this paper, UUV can dodge obstacles and threats, and search for the efficiency path.

## 1. Introduction

Unmanned underwater vehicles (UUV) were first designed for military purposes. Comparing it with an ordinary vehicle, it is more suitable for stealthy and scouting missions. Recent advances in technology have driven the development of unmanned vehicles to a new level. UUV are widely applied in civilian applications such as surveying, landscape mapping, and life rescuing [1]. A major challenge in the development of UUV is the realization of a real-time path planning and obstacle avoidance strategy that can effectively guide the vehicle in unstructured environment [2].

In recent years, many researchers have developed various approaches to solve the path planning problems, such as genetic algorithms (GAs), linear programming, potential fields, probabilistic sampling methods like rapidly exploring random trees (RRTs), and artificial intelligence (AI) methods like A*, which assures the path optimality. Cheng et al. [1] proposed a GA-inspired UUV path planner that is based on dynamic programming (DP). In the proposed path planner, the random-based crossover operator in GA is replaced with a deterministic crossover operator. The proposed path planner can always provide the best combination of crossover points from the available path segments. Li et al. [3] proposed a novel artificial bee colony (ABC) algorithm for unmanned combat aerial vehicles (UCAVs) path planning. Simulation results confirm that the algorithm is more competent for the UCAV path panning scheme than other ABC algorithms. Mansury et al. [4] have proposed a solution to the problem of path planning using artificial bee colony (ABC) algorithm and cubic Ferguson splines. Firstly, a path for robot movement is described by Ferguson splines and then ABC algorithm is used to optimize the parameter of splines to find an optimal path between the starting and the goal points considering obstacles between the starting and the goal points considering obstacles between them. Khelchandra and Jie [5] proposed a path planning method that is based on random sampling. Their proposed method has shown a high probability to find collision-free paths in short time. However, because of its randomized nature, their method may come up with infeasible solutions. Fern$\overset{´}{\text{a}}$ndez-Perdomo et al. [6] proposed a novel path planning algorithm for gliders using ocean currents. It bases on the A* family of algorithms and incorporates a probabilistic framework. Instead of discretizing the search space, a set of bearing angles is sampled at each surfacing point and the glider trajectory is integrated. Like other exact algorithms, the computational complexity of an A* algorithm increases with the size of the search domain. Li et al. [7] redefined potential functions to eliminate oscillations and local minima problems and use improved wall-following methods for the robots to escape nonreachable target problems. Meanwhile, they develop a regression search method to optimize the planned path. The optimization path is calculated by connecting the sequential points produced by improved artificial potential field (APF) method. The simulation results confirm that the proposed path planning approach can calculate a shorter or more nearly optimal path than the general APF. Jaillet et al. [8] presented a sampling-based algorithm to compute paths in problems which involve high-dimensional cost spaces. The proposed method combines the exploratory strength of the rapidly exploring random tree (RRT) algorithm, with the efficiency of stochastic optimization methods. It integrates an adaptive mechanism that helps to ensure a good performance for a large set of problems. Due to the nature of path planning, it is a constrained optimization problem, and many optimization algorithms have been proposed to overcome the problem. Brand et al. [9] proposed the application of ant colony optimization and compared two different pheromone reinitialization schemes.

Operating in an unknown semistructured environment, UUV may encounter obstacles which are not described in the electronic chart. In order to ensure global and real-time path planning, Yun et al. [10] divided the path planning algorithm into two levels: firstly, employing global path planning based on geometric method and then carrying out local path planning based on one new artificial field potential function, which uses sector as the parameter. In order to ensure real-time path planning, the information of sonar is combined to the static global map. Dolgov et al. [11] described a practical path-planning algorithm for an autonomous vehicle operating in an unknown semistructured environment, where obstacles are detected online by the robot's sensors. Firstly, they use a variant of A* search to obtain a kinematically feasible trajectory and then improve the quality of the solution via numeric nonlinear optimization, leading to a local optimum. Further, they extend algorithm to use prior topological knowledge of the environment to guide path planning, leading to faster search and final trajectories better suited to the structure of the environment.

UUV must have homing and docking functions in order to be completely autonomous. Homing is an operation in which UUV return to the vicinity of the launcher after a mission. Path planning and tracking control of the planned path are also included in this operation. Docking is another operation in which UUV are fixed with the launcher exactly when it is close to the launcher. Docking is more difficult than homing since the position of the launcher can be changed easily by ocean current and waves. Hence, much more precise path considered that future position of the launcher is needed [12]. Nowadays, many of the docking methods have been studied. But, most of the developed methods are concerned about towing or underwater structure designed for docking with the docking station [13, 14]. And many articles are concerned about providing accurate measurement of the UUV position and orientation or providing control strategy for UUV docking [15–18]. However, plan homing and docking path is the first step for UUV homing and docking. Sujit et al. [19] present a navigation function based approach for docking onto a moving submarine. The motion planning system is based on potential fields but avoids the problem of local minima around no fly zones by using a Koditschek and Rimon navigation function. The authors represented the no fly zones with circles and ellipses; these zones have tangential potential. Thus, when the AUV reaches them, it gets deflected away depending on the direction of the tangential potentials. Using these directional potentials of the no fly zones, the navigation function based controller must guide the AUV towards the dock. But the resulting path is not an optimal rendezvous.

In this paper, we proposed a strategy to plan UUV homing and docking path in movement disorders environment. And the application of the particle swarm optimization (PSO) algorithm is investigated [20, 21]. PSO is considered as one of the modern heuristic algorithms for optimization first proposed by Kennedy and Eberhart in 1995 [22]. The motivation for the development of this method was based on the simulation of simplified animal social behaviors [23]. The PSO algorithm works on the social behavior of particles in the swarm [24]. In PSO, the population dynamics simulates a bird flock's behavior where social sharing of information takes place and individuals can profit from the discoveries and previous experience of all other companions during the search for food. That is, the global best solution is found by simply adjusting the trajectory of each individual towards its own best location and towards the best particle of the entire swarm at each time step [22, 23, 25]. Owing to its reduction on memory requirement and computational efficiency with convenient implementation, it has gained lots of attention in various optimal control system applications, compared to other evolutionary algorithms  [20, 21, 26]. Several researches were carried out so far to analyze the performance of the PSO with different settings; for example, Shi and Eberhart [27] indicated that the optimal solution can be improved by varying the value of *ω*  from 0.9 at the beginning of the search to 0.4 at the end of the search for most problems, and they introduced a method named TVIW with a linearly varying inertia weight over the generations. Guimin et al. [28] introduced exponential inertia weight strategies, which is found to be very effective for TVIW. Ratnaweera et al. [23] propose time-varying acceleration coefficients as a parameter automation strategy for the PSO named TVAC, which reduces the cognitive component and increases the social component by changing the acceleration coefficients.

The contribution of this paper is concluded as follows. Firstly, the avoiding static obstacle cost function, the avoiding dynamic obstacle cost function, and the approaching objective cost function are built to construct the path planning cost function. Secondly, we proposed a new strategy to plan path for UUV homing and docking. Thirdly, we proposed a novel particle swarm optimization algorithm and used it to obtain the optimization cost function for path point.

The rest of this paper is organized as follows. In Section 2, the path planning cost function and a new strategy to plan path for UUV homing and docking are introduced. In Section 3, the basic PSO methodology and previous developments are summarized. In Section 4, a novel particle swarm optimization algorithm is proposed. In Section 5, the experimental settings for the benchmarks and simulation strategies are explained, and the conclusion is drawn based on the comparison analysis. In Section 6, the simulation of UUV path planning is explained. In Section 7, we present some concluding remarks.

## 2. UUV Path Planning for Homing and Docking

In this section, UUV path planning for mother vessel recovery using PSO in dynamic obstacles environment is presented and the reasonable path is studied. Path planning cost function has three parts: cost function for static obstacles, cost function for dynamic obstacles, and cost function for approaching target. To simplify the issue, UUV sail in the mission zones at certain fixed velocity so that UUV sail at certain fixed distance in each step. As each step for path planning has a fixed length of time, if we obtain the heading angle for UUV sailing to next waypoint, then we can obtain the position of the next waypoint. Set each particle of particle swarms as a heading angle for UUV sailing to next waypoint. Using NPSO, we can obtain the heading angle for UUV sailing to the optimal next waypoint with minimum cost function.

### 2.1. UUV Path Planning Cost Function

Firstly, define the initial position *P*_0_(*x*_0_, *y*_0_), the waypoint *P*_*i*_(*x*_*i*_, *y*_*i*_),  *i* = 1,2,…, *n*, and the target *P*_*g*_(*x*_*g*_, *y*_*g*_).

#### 2.1.1. Cost Function for Static Obstacles

**Figure 1:**
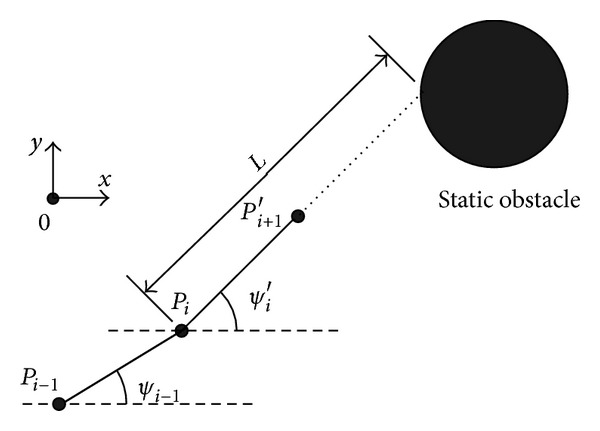
Schematic diagram of static obstacles avoidance for UUV.

In the underwater environment, UUV have to avoid static obstacles including submarine ridge, reef rock, and hidden pole. In Figure 1, static obstacles avoidance for UUV is illustrated, and the obstacles are represented by circles.

If the position of UUV is *P*_*i*_, the next candidate waypoint is *P*_*i*+1_′. As shown in Figure 1, the straight distance *L* is range from *P*_*i*_ to obstacle at the direction along *P*_*i*+1_′. Cost function for static obstacles can be written as

(1) $$\begin{array}{c} F_{1}=\frac{N*V}{L}, \end{array}$$

where *N* is an adjusting coefficient, which is constant and given. *V* is velocity of UUV, which is given. The shorter the time from UUV to obstacle is, the larger the value of *F*_1_ is, and vice versa. Set the candidate waypoint with minimum value of *F*_1_ as the next waypoint.

#### 2.1.2. Cost Function for Dynamic Obstacles

**Figure 2:**
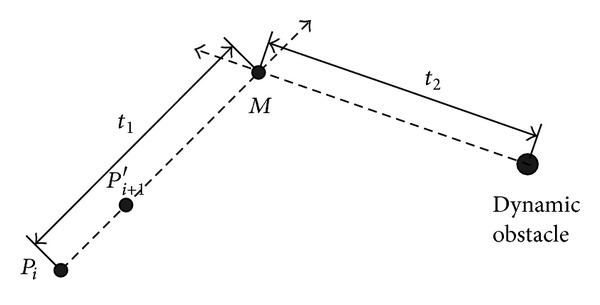
Schematic diagram of dynamic obstacles avoidance for UUV.

UUV also have to avoid dynamic obstacles existing in the mission zone. In Figure 2, the point *M* is regarded as the cross point of the line *P*_*i*_ *P*_*i*+1_′ and the movement direction for dynamic obstacle. Assume that the time interval of UUV moving from *P*_*i*_ to *M* is *t*_1_ and the time interval of dynamic obstacle motion to *M* is *t*_2_. If *t*_2_ > *t*_1_, it is said that UUV are faster than the obstacle to arrive at *M*. When *t*_2_ − *t*_1_ is large, UUV will safely sail. Choose the avoiding cost function *e* ^−*β*(*t*_2_−*t*_1_)^2^^. Besides, when the time between UUV and *M* is longer, more safety is obtained, and vice versa. Therefore, the cost function for dynamic obstacles is adjusted to

(2) $$\begin{array}{c} e^{-\alpha t_{1}^{2}}*e^{{-\beta (t_{2}-t_{1})}^{2}}. \end{array}$$

If *t*_2_ < *t*_1_, it is obvious that the dynamic obstacles will arrive at point *M* earlier than UUV, and UUV can avoid the dynamic obstacle safely. Above all, cost function for dynamic obstacles *F*_2_ is

(3) $$\begin{array}{c} F_{2}=\{\begin{array}{cc} e^{-[\alpha t_{1}^{2}+{\beta (t_{2}-t_{1})}^{2}]} & t_{2}>t_{1}\\ 0 & \text{others}. \end{array} \end{array}$$

Consider the size of UUV and dynamic obstacle, *F*_2_ in (3) can be rewritten as

(4) $$\begin{array}{c} F_{2}=\{\begin{array}{cc} e^{-[\alpha t_{1}^{2}+{\beta (t_{2}-t_{1}+\tau_{1})}^{2}]} & t_{2}>t_{1}-\tau_{2}\\ 0 & \text{others}, \end{array} \end{array}$$

where *α* and *β* are adjusting coefficients, which are constant and given, *τ*_1_ is the ratio of length to velocity of UUV, and *τ*_2_ is the ratio of length to velocity of dynamic obstacle. If the cross point *M* vanishes, UUV trajectory and the dynamic obstacle trajectory have no intersection, and *F*_2_ = 0.

#### 2.1.3. Cost Function for Approaching Target

**Figure 3:**
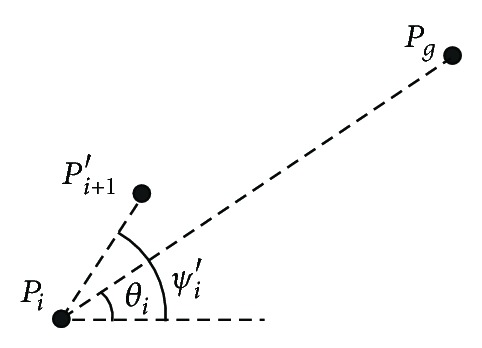
Schematic diagram of approaching target.

To implement the motion of UUV towards the target, cost function for approaching target is designed. The schematic diagram of approaching target is shown in Figure 3.

In Figure 3, *θ*_*i*_ describes the angle of the connecting line between *p*_*i*_ and *P*_*g*_. Because *p*_*i*_ and *P*_*g*_ are known, so it is easy to calculate the *θ*_*i*_. *ψ*_*i*_′ is optimization parameter, which represents the heading angle of candidate waypoint *P*_*i*+1_′. When *ψ*_*i*_′ = *θ*_*i*_, UUV move to target directly, and cost function for approaching target *F*_3_ is the strongest. So, normal distribution is introduced to represent *F*_3_. Consider

(5) $$\begin{array}{c} F_{3}=\frac{1}{\sqrt{2\pi }\sigma }e^{-{\left(\psi -\theta \right)}^{2}/2\sigma^{2}}. \end{array}$$

#### 2.1.4. UUV Path Planning Cost Function

UUV path planning cost function is iterated with three cost functions and described as

(6) $$\begin{array}{c} F=k_{1}F_{1}+k_{2}F_{2}-k_{3}F_{3}, \end{array}$$

where *k*_*i*_  is weight coefficients (*k*_*i*_ > 0), which are constant and given. UUV sail from initial waypoint with fixed velocity, and each step for path planning is fixed length of time. Set each particle of particle swarms as a heading angle for UUV sailing to the next waypoint. Using NPSO, we can obtain the heading angle for UUV sailing to the optimal next waypoint with minimum cost function *F*.

#### 2.1.5. The Admittance Angle for Homing and Docking

**Figure 4:**
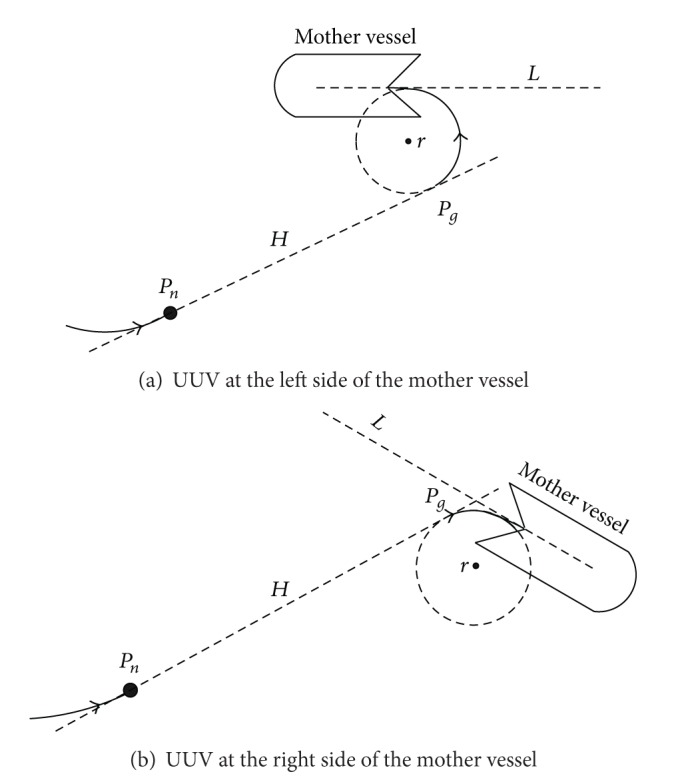
The recovery admittance angle.

For homing and docking, UUV will enter into the mother vessel (launcher) with a fixed angle (Figure 4). To obtain the effective path at the end of the planning path, let *L* denote mother vessel hatch center line and a circle is made with the radius *r* which is tangent to one point with the line *L*. The range of tangent point to the hatch is larger than the length of vessel (generally choose the length of two UUV), and *r* is larger than minimum turning radius.

In the path planning for homing and docking, the objective *p*_*g*_ is the tangent point between the circle and the line through the current UUV path point *P*_*n*_. When |*P*_*n*_ *P*_*g*_| < *vτ* (where *v* is the UUV velocity and *τ* is the time slot for one step), the path planning is finished and UUV enter into mother vessel at the end of arc.

Influenced by ocean current and waves, the mother vessel is moving, and UUV are moving and has to avoid obstacles, so UUV may sail change between the right side and the left side of the mother vessel. No matter what happened, we just make sure that the circle is in the same side of the mother vessel with UUV.

## 3. Some Previous Work of PSO

Introduced by Dr. Kennedy and Dr. Eberhart in 1995, PSO has ever since turned out to be a competitor in the field of numerical optimization, and there has been a considerable amount of work done in developing the original version of PSO. In this section, we summarize some entire significant previous developments.

### 3.1. Basic Particle Swarm Optimization (BPSO)

In PSO, each solution called a “particle” flies in the search space searching the optimal position to land. PSO system combines local search method (through individual experience) with global search methods (through neighboring experience), attempting to balance exploration and exploitation [29]. Each particle has a position vector *x*_*i*_(*k*), a velocity vector *v*_*i*_(*k*), the position with the best fitness encountered by the particle, and the index of the best particle in the swarm. The position vector and the velocity vector of the *i*th particle in the *d*-dimensional search space can be represented as *x*_*i*_ = (*x*_*i*1_, *x*_*i*2_, *x*_*i*3_,…*x*_*id*_) and *v*_*i*_ = (*v*_*i*1_, *v*_*i*2_, *v*_*i*3_,…, *v*_*id*_), respectively. The best position of each particle (*p*_best_) is *p*_*i*_ = (*p*_*i*1_, *p*_*i*2_, *p*_*i*3_,…, *p*_*id*_), and the fitness particle found so far at generation *k* (*g*_best_) is *p*_*g*_ = (*p*_*g*1_, *p*_*g*2_,…, *p*_*gd*_). In each generation, each particle is updated by the following two equations:

(7) vid(k+1)=vid(k)+c1×r1×(pid(k)−xid(k)) +c2×r2×(pgd(k)−xid(k)),

(8) $$\begin{array}{c} x_{id}\left(k+1\right)=x_{id}\left(k\right)+v_{id}\left(k+1\right). \end{array}$$

The parameters *c*_1_ and *c*_2_ are constants known as acceleration coefficients. *r*_1_ and *r*_2_ are random values in the range from 0 to 1, and the value of *r*_1_ and *r*_2_ is not the same for every iteration. Kennedy and Eberhart [22] suggested setting either of the acceleration coefficients at 2, in order to make the mean of both stochastic factors in (7) unity so that particles would over fly only half the time of search. The first equation shows that, in PSO, the search towards the optimum solution is guided by the previous velocity, the cognitive component, and the social component.

Since the introduction of the particle swarm optimization, numerous variations of the algorithm have been developed in the literature. Eberhart and Shi showed that PSO searches for wide areas effectively but tends to lack local search precision. They proposed in that work a solution by introducing *ω*, an inertia factor. In this paper, we name it as basic particle swarm optimization (BPSO):

(9) vid(k+1)=ω×vid(k)+c1×r1×(pid(k)−xid(k)) +c2×r2×(pgd(k)−xid(k)),xid(k+1)=xid(k)+vid(k+1).

### 3.2. Time-Varying Inertia Weight (TVIW)

The role of the inertia weight *ω*  is considered very important in PSO convergence behavior. The inertia weight is applied to control the impact of the previous history of velocities on the current velocity. Large inertia weight facilitates global exploration, while small one tends to facilitate local exploration. In order to assure the particles converge to the best point in the course of the search, Shi and Eberhart [30] have found that time-varying inertia weight (TVIW) has a significant improvement in the performance of PSO and proposed linear decreasing inertia weight PSO (LWPSO) with a linear decreasing value of *ω*. This modification can increase the exploration of the parameter space during the initial search iterations and increase the exploitation of the parameter space during the final steps of the search [31]. The mathematical representation of inertia weight is given as follows:

(10) $$\begin{array}{c} \omega =\left(\omega_{1}-\omega_{2}\right)\times \left(\frac{\text{MAXITER}-k}{\text{MAXITER}}\right)+\omega_{2}, \end{array}$$

where *ω*_1_ and *ω*_2_ are the initial and final values of the inertia weight, respectively, *k* is the current iteration number, and MAXITER is the maximum number of allowable iterations. Shi and Eberhart [27] indicate that the optimal solution can be improved by varying the value of *ω* from 0.9 at the beginning of the search to 0.4 at the end of the search for most problems.

Guimin et al. [28] proposed natural exponential (base *e*) inertia weight strategies, named EPSO and expressed as

(11) $$\begin{array}{c} \omega =\omega_{2}+\left(\omega_{1}-\omega_{2}\right)\times \exp\left[-{\left(\frac{K}{\left(\text{MAXITER}/4\right)}\right)}^{2}\right]. \end{array}$$

### 3.3. Time-Varying Acceleration Coefficient (TVAC)

In PSO, the particle updated due to the cognitive component and the social component. Therefore, proper control of these two components is very important to find the optimum solution accurately and efficiently. Ratnaweera et al. [23] introduced a time-varying acceleration coefficient (TVAC), which reduces the cognitive component and increases the social component, by changing the acceleration coefficients *c*_1_ and *c*_2_ with the time evolution. The objective of this development is to enhance the global search in the early part of the optimization and to encourage the particles to converge towards the global optima at the end of the search. The TVAC is represented using the following equations:

(12) $$\begin{array}{c} c_{1}=\left(c_{\max}-c_{\min}\right)\frac{k}{\text{MAXITR}}+c_{\min},\\ c_{2}=\left(c_{\min}-c_{\max}\right)\frac{k}{\text{MAXITR}}+c_{\max}, \end{array}$$

where *c*_min⁡_ and *c*_max⁡_ are constants, *k* is the current iteration number, and MAXITR is the maximum number of allowable iterations.

Simulations were carried out with numerical benchmarks to find out the best ranges of values for *c*_1_ and *c*_2_. From the results, it was observed that the best solutions were determined when changing *c*_1_ from 2.5 to 0.5 and changing *c*_2_ from 0.5 to 2.5 over the full search range.

## 4. Proposed New Developments

In the particle swarm algorithm, the trajectory of each individual in the search space is adjusted by dynamically altering the velocity of each particle, according to its own flying experience and the flying experience of the other particles in the search space [23]. Kennedy and Eberhart [22] indicate that a relatively high value of the cognitive component, compared with the social component, will result in excessive wandering of individuals through the search space. In contrast, a relatively high value of the social component may lead particles to rush prematurely towards a local optimum.

Considering those concerns, a novel and effective approach to PSO algorithms is proposed in this paper. Particles are divided into several groups, and each group contains two particles. One particle in the group is noted as number 1, which is developed according to its own flying experience and the flying experience of swarms, and owns high individual experience acceleration coefficients and low group experience acceleration coefficients. So number 1 is encouraged to wander through the entire search space, without clustering around local optima, while the other particle in the group named number 2 is developed according to the flying experience of swarms and the flying experience of the group (both number 1 and number 2). When the flying experience of the group changed, set the flying experience of the group as position of number 2 and velocity of number 2 which is vanishing. Number 2 is encouraged to converge towards the global optima, with a small cognitive component and a large social component. Consider

(13) v1dt(k+1)=ω1tv1d(k)+cmax⁡r1t(p1dt(k)−x1dt(k)) +cmin⁡r2t(pgd(k)−x1dt(k)),v2dt(k+1) ={ω2tv2d(k) +cmin⁡r3t(pudt(k)−x2dt(k))  +cmax⁡r4t(pgd(k)−x2dt(k))pudt(k)≥pudt(k−1)0pudt(k)<pudt(k−1),x1dt(k+1)=x1dt(k)+v1dt(k+1),x2dt(k+1) ={x2dt(k)+v2dt(k+1)pudt(k)≥pudt(k−1)pudt(k)pudt(k)<pudt(k−1),

where *r*_1_^*t*^, *r*_2_^*t*^, *r*_3_^*t*^, and *r*_4_^*t*^ are random values, uniformly distributed between zero and one, and the their value is not same for every iteration, *x*_*id*_^*t*^(*k*) is the *d*th dimension position of the subparticle *i* of the *t*th group after *k* time iteration, *ω*_*i*_^*t*^ is the inertia weigh value of the subparticle *i* of the *t*th group, *c*_max⁡_ is the maximum value of acceleration coefficients, *c*_min⁡_ is the minimum value of acceleration coefficients, *v*_*id*_^*t*^(*k*) is the *d*th dimension velocity of the subparticle *i* of the *t*th group during *k* time iteration, *p*_*id*_^*t*^(*k*) is the *d*th dimension position of the optimal position of the *i*th subparticle of the *t*th groupafter *k* time iteration, *p*_*ud*_^*t*^(*k*) is the *d*th dimension position of the optimal position of the *t*th group after *k* time iteration, and *p*_*gd*_(*k*) is the *d*th dimension position of the swarm optimal position after *k* time iteration.

**Remark 1 .**

In this paper, two subparticles are generated for each group; therefore, *i* = 1,2.

Define *n* as the number of particles in the swarm, *M*  as the maximum iteration frequency, and fitness (*x*_*i*_^*t*^(*k*)) as the value of the cost function for the subparticle *i*  of the *t*th group after *k* time iteration.

The detailed steps are shown as in Algorithm 1.

## 5. Experimental Settings and Simulation for Benchmark Testing

Simulations were carried out to observe the rate of convergence and the quality of the optimum solution of the new methods introduced in this investigation by comparing between BPSO, LWPSO, EPSO, and TVAC. From the standard set of benchmark problems available in the literature, there are 6 important functions considered to test the efficacy of the proposed method. All of the test functions reflect different degrees of complexity.

### 5.1. Functions Introduction

The functions are as follows.

#### 5.1.1. Sphere Function

Consider

(14) $$\begin{array}{c} f_{1}\left(x\right)=\overset{D}{\underset{i=1}{\sum }}x_{i}^{2}. \end{array}$$

With the search space {*x*_*i*_∣ − 100 < *x*_*i*_ < 100}, the global minimum is located at *x* = [0,…,0]^*D*^ with *f*(*x*) = 0. It is very simple, convex unimodal function with only one local optimum value.

#### 5.1.2. Rotated Hyperellipsoid Function: Schwefel's Problem 1.2

Consider

(15) $$\begin{array}{c} f_{2}\left(x\right)=\overset{D}{\underset{i=1}{\sum }}{\left(\overset{i}{\underset{j=1}{\sum }}x_{j}\right)}^{2}. \end{array}$$

With the search space {*x*_*i*_∣ − 100 < *x*_*i*_ < 100}, the global minimum is located at *x* = [0,…,0]^*D*^ with *f*(*x*) = 0. It is continuous, convex, and unimodal. With respect to the coordinate axes, this function produces rotated hyperellipsoids.

#### 5.1.3. Rosenbrock Function

Consider

(16) $$\begin{array}{c} f_{3}\left(x\right)=\overset{D-1}{\underset{i=1}{\sum }}\left[100{\left(x_{i+1}-x_{i}^{2}\right)}^{2}+{\left(x_{i}-1\right)}^{2}\right]. \end{array}$$

With the search space {*x*_*i*_∣ − 100 < *x*_*i*_ < 100}, the global minimum is located at *x* = [1,…,1]^*D*^ with *f*(*x*) = 0. It is a unimodal function and the global optimum is inside a long, narrow, parabolic shaped flat valley. Finding the valley is trivial.

#### 5.1.4. Rastrigin Function

Consider

(17) $$\begin{array}{c} f_{4}\left(x\right)=\overset{D}{\underset{i=1}{\sum }}\left[x_{i}^{2}-10\cos\left(2\pi x_{i}\right)+10\right]. \end{array}$$

With the search space {*x*_*i*_∣ − 100 < *x*_*i*_ < 100}, the global minimum is located at *x* = [0,…,0]^*D*^ with *f*(*x*) = 0. It is highly multimodal. However, the locations of the minima are regularly distributed.

#### 5.1.5. Griewank Function

Consider

(18) $$\begin{array}{c} f_{5}\left(x\right)=\frac{1}{4000}\overset{D}{\underset{i=1}{\sum }}x_{i}^{2}-\overset{D}{\underset{i=1}{\prod }}\cos\left(\frac{x_{i}}{\sqrt{i}}\right)+1. \end{array}$$

With the search space {*x*_*i*_∣ − 100 < *x*_*i*_ < 100}, the global minimum is located at *x* = [0,…,0]^*D*^ with *f*(*x*) = 0. It is a multimodal function and has many widespread local minima. However, the locations of the minima are regularly distributed.

#### 5.1.6. Sum of Different Powers Function

Consider

(19) $$\begin{array}{c} f_{6}\left(x\right)=\overset{D}{\underset{i=1}{\sum }}{\left|x_{i}\right|}^{\left(i+1\right)}. \end{array}$$

The sum of different powers is a commonly used unimodal test function. With the search space {*x*_*i*_∣ − 100 < *x*_*i*_ < 100}, the global minimum is located at *x* = [0,…, 0]^*D*^ with *f*(*x*) = 0.

### 5.2. The Coefficients Setting

The parameters for simulation are listed in the Table 1.

In this table, *c*_(·)_ expresses the accelerations coefficients, *ω* denotes inertia weight, the dimension is *D*, and the range of the search space and the velocity space are *x*(·) and *v*(·). If the current position is out of the search space, the position of the particle is taken to be the value of the boundary and the velocity is taken to be zero. If the velocity of the particle is outside of the boundary, its value is set to be the boundary value. The maximum number of iterations is set to 1000. For each function, 100 trials were carried out and the average optimal value and the standard deviation are presented. To verify the performance of the algorithm at different dimensions, variable dimension *D*  increases from 10 to 100, and the optimal mean and variance of test function with 6 algorithms are calculated.

### 5.3. The Results Comparing NPSO with the Previous Developments of PSO

The simulation results are given in Table 2. The comparison results elucidate that the searching accuracy and stability arranged from low to high are BPSO, LWPSO, EPSO, TVAC, and NPSO for unimodal function. For multimodal function, the performance of NPSO is the same as TVAC, but, for the highly multimodal Rastrigin function, TVAC is superior to NPSO.

**Figure 5:**
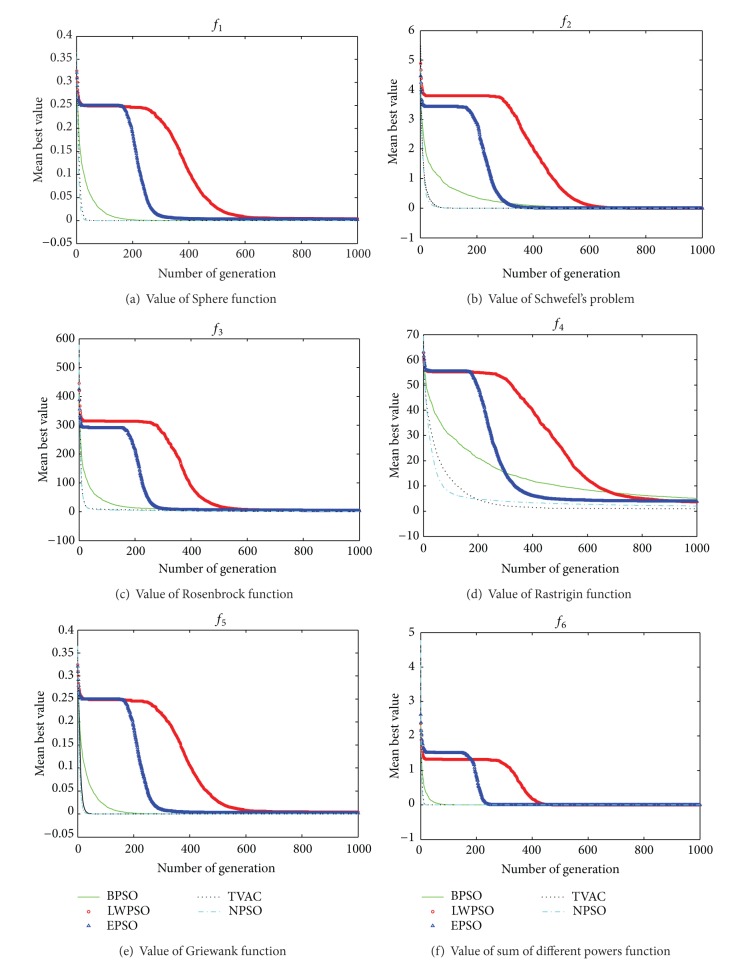
Variation of average optimum value with time.

With the increase of test functions dimension, the searching accuracy and stability for each algorithm are decreased. The performance of NPSO is superior to other algorithms. With regard to high multimodal function, the performance of TVAC is superior to NPSO. From Figure 5, it shows that the order of searching speed from high to low is BPSO (green solid curve), LWPSO (red circle), EPSO (blue triangle), TVAC (black dot curve), and NPSO (blue dash-dot). It is obvious that the performance of NPSO is more effective than the other algorithms. This is because, for the NPSO, two-particle coordination evolution is in the same group. One particle of the group is encouraged to wander through the entire search space, without clustering around local optima. The other particle of the group is flying between group experiences and swarm experiences, which is encouraged to converge towards the global optima.

## 6. The Simulation of UUV Path Planning

**Figure 6:**
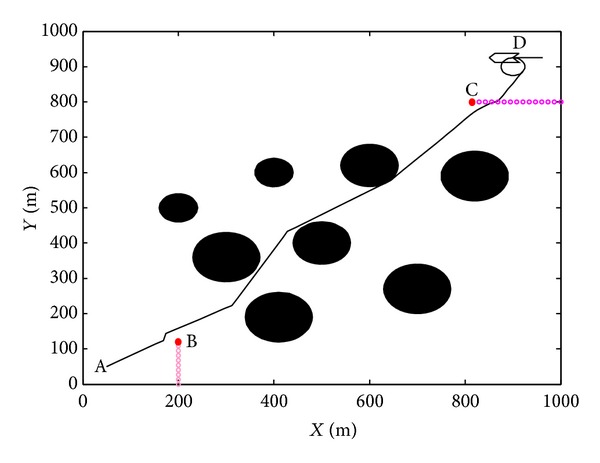
Path planning in dynamic obstacles environment.

The simulation is designed using the proposed algorithm for UUV path planning in dynamic obstacles environment. UUV start at the initial point, and the candidate point at the next step is searched using NPSO in the range of the turning angle limitation. And the next path point is obtained when the cost function reaches minimum value. Simulation is designed in the map with size of 1000 m × 1000 m in Figure 6. The initial point is A (50, 50), and dock hatch point is D (900, 900). The maximum turning limitation is |Δ*ψ*| ≤ *π*/3, *k*_1_ = *k*_2_ = *k*_3_ = 1, *N* = 1. To simplify the issue, the velocity is set to a constant 4 m/s, and UUV will encounter dynamic obstacle B which is parallel to the *y*-axis with sway coordinate at 200 m and dynamic obstacle C parallel to the *x*-axis with surge coordinate at 800 m.

In Figure 6, for obstacle B, UUV avoid it by steering left. For obstacle C, UUV avoid it by changing its course towards its starboard side. The simulation results demonstrate that the proposed algorithm is effective to implement the path planning in dynamic obstacle environment.

To compare the performance of NPSO with LWPSO/EPSO/TVAC for path planning, the simulation is designed for path planning without dynamic obstacles.  Table 3 shows the parameters that were used for the different algorithms used, and Table 4 shows the simulation results.

From Table 4, NPSO and TVAC present better performance than the other PSO.

**Figure 7:**
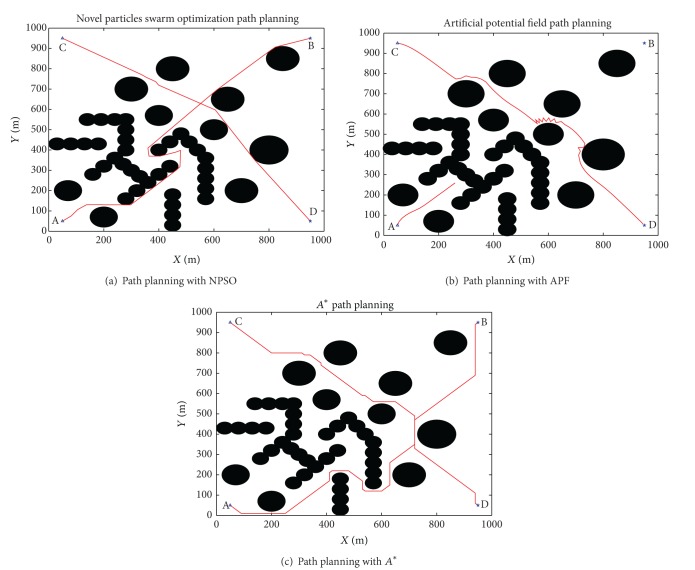
UUV path planning under complex environment.

To compare the performance of the algorithm proposed in this paper with traditional APF and A*, complex environment map has been introduced in Figure 7.

As shown in Figure 7, it turns out that proposed algorithm can obtain a better performance than the traditional APF for UUV path planning in complex environment. If the starting point is A (50, 50), and the target is B (950, 950), the traditional APF is easy to fall into local minima and cannot finish the path planning. To further analyze the performance, we set starting point C (50, 950) and target D (950, 50). From Figure 7, traditional APF is easy to shock in front of the obstacle. The A* algorithm discretizes the search space with a uniform grid, so the possible bearings are discretized too. As a consequence, the time between consecutive surfacing is nonconstant. From Table 5, the algorithm proposed can obtain shorter path than the other algorithms. The proposed algorithm can obtain a more smooth path and is superior to traditional APF and A* algorithm.  Table 6 shows the computation time of different algorithms. It is clear that traditional APF spent shorter time than other algorithms, and NPSO spent the longest time.

## 7. Conclusion

In this paper, UUV path planning for homing and docking in dynamic obstacle environment using NPSO is present and the appropriate strategy is explored at the mother vessel recovery. The simulation results demonstrate the feasibility of NPSO. UUV can avoid the dynamic obstacles and navigate along the reasonable path to implement the recovery.

The simulation of UUV path planning in complex environment shows that the proposed algorithm can get better path than traditional APF and A* algorithm, and it is not easy to be trapped in local minima. In order to completely escape from local minima or U shaped obstacles, it is necessary to design some escape rules, such as wall-following, random escaping.

NPSO is introduced and tested through a set of 6 benchmark functions and the statistical analyses of the simulated results are compared with BPSO, LWPSO, EPSO, and TVAC. The test of proposed method with the unimodal benchmark functions indicates its superiority over the other methods. However, for highly multimodal Rastrigin function, TVAC shows better performance than NPSO.

## Figures and Tables

**Algorithm 1:**
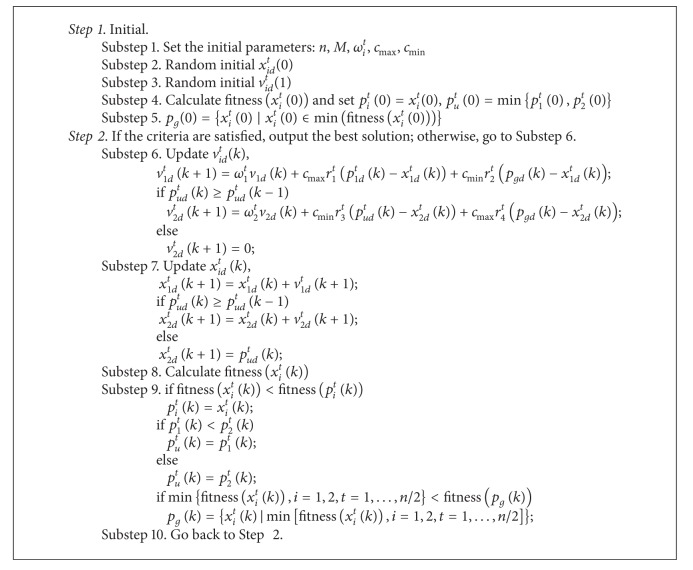
Algorithm 1

**Table 1:** Parameters for simulation.

Method	Parameters	
NPSO	ω_*i*_^*t*^ = 0.7, *c*_max⁡_ = 2.5, *c*_min⁡_ = 0.5	*n* = 30, *D* = 10, *x*_*i*_ ∈ [−100,100], *v*_*i*_ ∈ [−100,100]
BPSO	*c*_1_ = *c*_2_ = 2.0, *ω* = 0.7	
LWPSO	*c*_1_ = *c*_2_ = 2.0, ω_1_ = ω_max⁡_ = 0.9, ω_2_ = ω_min⁡_ = 0.4	
EPSO	*c*_1_ = *c*_2_ = 2.0, ω_1_ = ω_max⁡_ = 0.9, ω_2_ = ω_min⁡_ = 0.4	
TVAC	*c*_max⁡_ = 2.5, *c*_min⁡_ = 0.5, *ω* = 0.7	

**Table 2:** Comparison between BPSO, LWPSO, EPSO, TVAC, and NPSO.

*F*	*D*	Average (standard deviation)				
		BPSO	LWPSO	EPSO	TVAC	NPSO
*f*_1_	10	4.7979*e* − 11 (1.1343*e* − 10)	3.2219*e* − 24 (1.0706*e* − 23)	8.7747*e* − 51 (3.408*e* − 50)	1.4826*e* − 54 (1.4770*e* − 53)	2.2681*e* − 78 (2.1874*e* − 77)
	30	5.3768 (4.3459)	4.0137*e* − 05 (5.926*e* − 05)	2.3219*e* − 12 (1.1495*e* − 11)	1.1062*e* − 10 (7.3302*e* − 10)	2.0175*e* − 23 (2.0172*e* − 22)
	50	2.8412*e* + 01 (4.5047)	0.2149 (0.1634)	2.2215*e* − 05 (3.5696*e* − 05)	0.0019 (0.0079)	7.6676*e* − 09 (5.7490*e* − 08)
	70	4.4685*e* + 01 (4.3454)	1.1727*e* + 01 (1.2113*e* + 01)	0.0192 (0.0238)	0.0609 (0.1033)	0.0010 (0.0082)

*f*_2_	10	0.0013 (0.0018)	4.0624*e* − 08 (1.10659*e* − 07)	3.1542*e* − 15 (1.9686*e* − 14)	3.6072*e* − 27 (1.6376*e* − 26)	3.1897*e* − 27 (1.0275*e* − 26)
	30	2.0647*e* + 01 (7.9831)	3.7596 (2.0255)	0.5809 (0.3373)	0.0091 (0.0197)	0.0012 (0.0101)
	50	7.2982*e* + 01 (2.8263*e* + 01)	2.6189*e* + 01 (1.3125*e* + 01)	1.0295*e* + 01 (5.1265)	0.4883 (0.3021)	0.1684 (0.7178)
	70	1.4821*e* + 02 (4.6281*e* + 01)	6.9336*e* + 01 (3.3717*e* + 01)	2.9336*e* + 01 (1.0417*e* + 01)	2.7039 (1.0853)	0.8808 (1.3369)

*f*_3_	10	5.6418 (1.092)	4.3037 (1.2401)	3.1701 (1.2637)	0.7262 (0.9490)	0.6790 (1.0522)
	30	1.2322*e* + 03 (9.4693*e* + 02)	4.2313*e* + 01 (2.6596*e* + 01)	3.1052*e* + 01 (1.7699*e* + 01)	2.3370*e* + 01 (1.9238)	2.2096*e* + 01 (5.8478)
	50	6.5575*e* + 03 (1.3919*e* + 03)	2.9054*e* + 02 (1.6229*e* + 02)	7.3894*e* + 01 (3.6633*e* + 01)	4.6563*e* + 01 (2.5861)	4.6325*e* + 01 (1.2231*e* + 01)
	70	1.1834*e* + 04 (1.9184*e* + 03)	5.2635*e* + 03 (4.0246*e* + 03)	2.0414*e* + 02 (7.4873*e* + 01)	7.5240*e* + 01 (1.2063*e* + 01)	8.0415*e* + 01 (2.9457*e* + 01)

*f*_4_	10	5.1090 (3.0667)	3.6806 (1.7685)	3.8703 (1.7882)	0.9750 (0.9693)	2.0911 (1.2360)
	30	1.5696*e* + 02 (3.9880*e* + 01)	3.2291*e* + 01 (1.0059*e* + 01)	2.9948*e* + 01 (7.1988)	1.7282*e* + 01 (5.4892)	2.0219*e* + 01 (6.9868)
	50	3.8006*e* + 02 (3.5258*e* + 01)	7.5142*e* + 01 (2.5871*e* + 01)	6.1896*e* + 01 (1.5107*e* + 01)	3.6575*e* + 01 (9.4137)	4.3956*e* + 01 (1.1176*e* + 01)
	70	5.8512*e* + 02 (3.6592*e* + 01)	1.6259*e* + 02 (6.2498*e* + 01)	8.8994*e* + 01 (1.7553*e* + 01)	5.4198*e* + 01 (1.1482*e* + 01)	7.3153*e* + 01 (1.4861*e* + 01)

*f*_5_	10	9.8646*e* − 05 (0.0009)	0.0041 (0.0096)	0.0017 (0.0055)	0 (0)	0 (0)
	30	0.1366 (0.1090)	0.0008 (0.0045)	0.0013 (0.0071)	1.0210*e* − 10 (1.0021*e* − 09)	2.3425*e* − 16 (8.3751*e* − 16)
	50	0.5856 (0.0891)	0.0027 (0.0021)	9.9034*e* − 05 (0.0009)	0.0001 (0.0007)	4.3243*e* − 10 (2.4063*e* − 09)
	70	0.6941 (0.0646)	0.0329 (0.0190)	0.0002 (0.0010)	0.0011 (0.0017)	1.1807*e* − 05 (4.5670*e* − 05)

*f*_6_	10	5.8516*e* − 18 (2.5897*e* − 17)	2.2299*e* − 40 (1.6366*e* − 39)	2.0832*e* − 84 (1.6585*e* − 83)	2.5229*e* − 87 (1.1045*e* − 86)	1.6901*e* − 96 (1.6901*e* − 95)
	30	1.9895*e* + 02 (6.2708*e* + 02)	1.0418*e* − 06 (3.7845*e* − 06)	7.3163*e* − 18 (2.7837*e* − 17)	1.0483*e* − 39 (7.2123*e* − 39)	4.6820*e* − 50 (4.6820*e* − 49)
	50	1.9927*e* + 07 (7.1603*e* + 07)	1.8348*e* + 01 (4.8704*e* + 01)	0.0017 (0.0067)	6.5668*e* − 25 (3.1648*e* − 24)	4.3136*e* − 21 (4.2561*e* − 20)
	70	1.5637*e* + 13 (8.3656*e* + 13)	2.6886*e* + 08 (1.6837*e* + 09)	1.4973*e* + 04 (1.0807*e* + 05)	4.9509*e* − 19 (2.6306*e* − 18)	7.7603*e* − 13 (7.5801*e* − 12)

**Table 3:** Parameters for simulation.

Method	Parameters	
NPSO	ω_*i*_^*t*^ = 0.7, *c*_max⁡_ = 2.5, *c*_min⁡_ = 0.5	*n* = 30, *D* = 3, *x*_*i*_ ∈ [−π/3, π/3], *v*_*i*_ ∈ [−π/3, π/3]
BPSO	*c*_1_ = *c*_2_ = 2.0, *ω* = 0.7	
LWPSO	*c*_1_ = *c*_2_ = 2.0, ω_1_ = ω_max⁡_ = 0.9, ω_2_ = ω_min⁡_ = 0.4	
EPSO	*c*_1_ = *c*_2_ = 2.0, ω_1_ = ω_max⁡_ = 0.9, ω_2_ = ω_min⁡_ = 0.4	
TVAC	*c*_max⁡_ = 2.5, *c*_min⁡_ = 0.5, *ω* = 0.7	

**Table 4:** Comparison between BPSO, LWPSO, EPSO, TVAC, and NPSO for UUV path planning.

*M*	Length of the path (m)				
	BPSO	LWPSO	EPSO	TVAC	NPSO
10	1290.2548	1289.9196	1289.4264	1288.8112	1288.0532
50	1287.8672	1287.8556	1287.8548	1287.8544	1287.8544
100	1287.8596	1287.8544	1287.8544	1287.8544	1287.8544
200	1287.8544	1287.8544	1287.8544	1287.8544	1287.8544
500	1287.8544	1287.8544	1287.8544	1285.7200	1284.8792
1000	1287.8544	1287.8544	1287.8544	1285.7200	1284.8792

**Table 5:** Comparison of different algorithms for path planning.

Path	Length of the path (m)		
	NPSO	APF	A∗
*A* → B	1593.79	—	1716.81
C → D	1307.15	1547.84	1401.66

**Table 6:** Computation time of different algorithms for path planning.

Path	Computation time (s)		
	NPSO	APF	A∗
*A* → B	94.83	—	11.70
C → D	54.55	0.27	10.17
